# A Survey of Teleceptive Sensing for Wearable Assistive Robotic Devices

**DOI:** 10.3390/s19235238

**Published:** 2019-11-28

**Authors:** Nili E. Krausz, Levi J. Hargrove

**Affiliations:** 1 Neural Engineering for Prosthetics and Orthotics Lab, Center of Bionic Medicine, Shirley Ryan AbilityLab (Formerly Rehabilitation Institute of Chicago), Chicago, IL 60611, USA; l-hargrove@northwestern.edu; 2 Biomedical Engineering Department, Northwestern University, Evanston, IL 60208, USA; 3 Physical Medicine and Rehabilitation Department, Northwestern University, Evanston, IL 60208, USA

**Keywords:** assistive robotics, rehabilitation robotics, teleceptive sensing, environment, computer vision, depth sensing, prostheses, exoskeletons

## Abstract

Teleception is defined as sensing that occurs remotely, with no physical contact with the object being sensed. To emulate innate control systems of the human body, a control system for a semi- or fully autonomous assistive device not only requires feedforward models of desired movement, but also the environmental or contextual awareness that could be provided by teleception. Several recent publications present teleception modalities integrated into control systems and provide preliminary results, for example, for performing hand grasp prediction or endpoint control of an arm assistive device; and gait segmentation, forward prediction of desired locomotion mode, and activity-specific control of a prosthetic leg or exoskeleton. Collectively, several different approaches to incorporating teleception have been used, including sensor fusion, geometric segmentation, and machine learning. In this paper, we summarize the recent and ongoing published work in this promising new area of research.

## 1. Introduction

Over the last few decades, the field of physical medicine and rehabilitation has seen a proliferation of wearable robotic devices designed to improve mobility, function, and quality of life for individuals with physical impairments. These wearable robots include powered prosthetic arms and legs [1,2,3,4] for individuals with amputations and powered exoskeletons and orthoses for individuals with paresis, paralysis, and other impairments [5,6]. Although numerous prototypes of each class of assistive or rehabilitative robot exists, such devices are not yet extensively used outside of the laboratory. Translation of wearable robots from the lab to the clinic and outside world is limited by several factors, including the increased metabolic cost of using these devices and difficulty in donning and doffing them, as well as their lack of robustness, limited functionality, and poor controllability [7]. Clearly each of these issues must be resolved, and each requires extensive study. In this review, we will focus on the limitations of current control systems that lead to poor controllability and limited functionality.

Awareness of, and the ability to respond to, perturbations and changes in the environment are essential for both living organisms and robots; however, these key elements are lacking from current control systems for assistive and rehabilitative robotic devices. Teleception can provide important environmental or contextual awareness for robust control systems that emulate the innate control systems of the human body.

Here we present a thorough survey of the different teleceptive technologies currently in existence, their benefits and limitations, and early use cases that have been proposed for wearable assistive robots. We discuss the results of these early efforts and possible directions for future research in this emerging field.

## 2. Teleceptive Sensing

Emulating the way the nervous system controls limbs could be the key to developing devices that operate effectively during locomotion and manipulation tasks. Control of movement in humans is regulated by two pathways: the descending pathways from the cerebellum and motor cortex that provide movement plans and patterns for the limbs, and the ascending pathways that obtain information from the senses and then return that information to the brain to modulate movement [8]. The structure of the intact human nervous system as an ideal control system for locomotion or manipulation then can be modeled by two primary elements: a feedforward model, and a feedback mechanism. In a control system, a feedforward model is defined by some control law or previously determined model, and feedback from sensors is used to adjust the feedforward model to improve movement [9]. In the case of the human model, sensor feedback can include proximate and remote, or teleceptive, sensing modalities.

Teleception is defined as sensing that occurs remotely, or with no physical contact being made with the object being sensed [10]. Humans have several teleceptive senses, including sight, hearing, and smell, in addition to the proximate senses of touch, taste, and proprioception. Some animals use other modalities of teleception, such as echolocation (bats) or magnetoreception (electric fish). In the case of locomotion or manipulation, teleceptive senses are primarily useful for developing an understanding of the environment, while proximate senses primarily provide an understanding of the physical movement of the person, though of course there are exceptions, such as using touch to interact with objects or the environment.

Effective control systems should include both proximate and teleceptive sensors, so that both the state of the environment and the subject within it can be integrated with the feedforward model to improve movement accuracy. For instance, when an individual is blindfolded during a locomotion task, they try compensate and gain an understanding of their environment and position in space by reaching out with their hands and moving their feet slowly over the ground to feel for any discontinuities.

**Figure 1:**
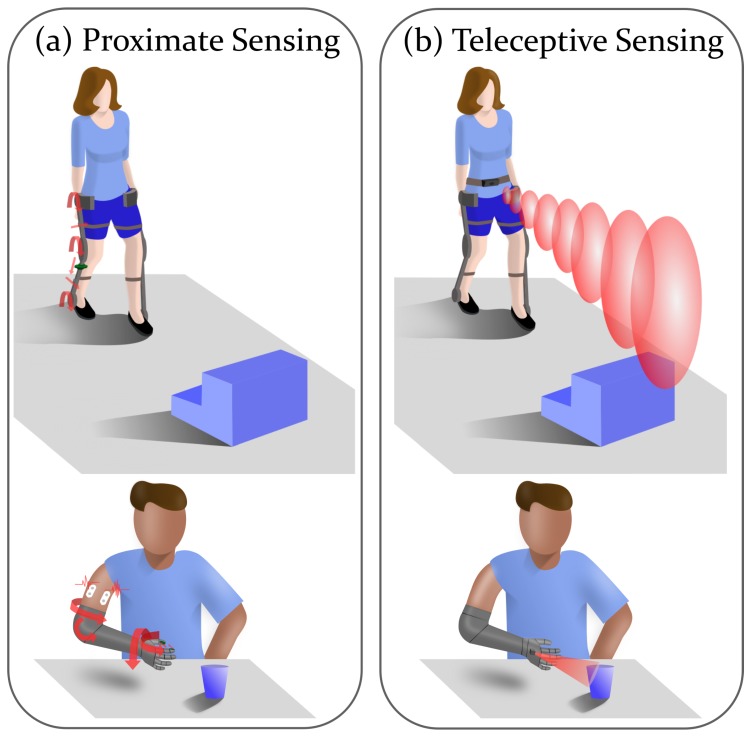
Wearable Assistive Devices using (**a**) Proximate Sensing or (**b**) Teleceptive Sensing. For each type of sensing, an illustration of a lower-limb orthosis (bottom) and an upper-limb prosthesis (top) using this sensor type is shown. Proximate sensing includes any sensor that directly measures the behavior of the individual or the assistive device, such as EMG sensors, load cells, encoders or potentiometers in motors, or IMUs. Teleceptive sensing includes any sensor that indirectly measures the environment or behavior of things external to the user, such as an RGB camera, IR sensor, Ultrasonic sensor, or Radar.

Adding environmental awareness to wearable robots may not only enhance the safety of the user by enabling them to respond to threats and navigate through obstacles, but may also make it possible to move from a reactive system (i.e., one that can only respond to immediate stimuli) to a system that can plan (i.e., one that can simulate responses to stimuli further away, with a longer timescale) [11]. An extended response time allows for additional computation and use of more complex prediction algorithms without lag or timing issues. Awareness of the environment can also shift the control burden from the user onto the robot, using semi- or fully autonomous control [12]. Adding teleceptive sensing to the control of prostheses and exoskeletons (as shown in Figure 1) can bridge the gap between robotics and assistive technology, and allow exploration of how users interact with their devices and how they can optimally share control with a robot. Hence the recent proliferation of work (as will be detailed later in this paper) to address the lack of teleception in assistive and rehabilitation devices.

## 3. Teleceptive Sensing Technology

Recently, development of semi- and fully autonomous vehicles [13,14] that use environmental sensing (primarily depth sensors) to locate obstacles in their path and predict the desired mode of operation, with little to no operator supervision, has accelerated [15,16]. This approach has been used in robotics [17], commercial products [18], and powered wheelchairs [19]. However, we believe that until recently, teleception has not been feasible or practical to use in control systems for powered prostheses or orthoses due to several factors, in particular, the high cost, large size, and lack of portability of teleceptive sensors and the difficulty of integrating them into devices. However, as technology has improved, new small, lightweight sensors in a variety of teleceptive modalities have become commercially available and inexpensive [20]. Simultaneously, advances in computing allowing for implementation of these approaches without massive computation requirements have made use of these sensors more realistic for integration into a wearable assistive device. In this review, we will consider sensor modalities such as RGB cameras, which sense the environment using visible light; infrared lasers or radar that use other electromagnetic radiation; and ultrasonic or sonar sensors that use sound waves.

### 3.1. Sensor Specifications

Existing teleceptive sensor technologies vary in size, robustness to lighting variation, geometric and temporal resolution, etc. To select the appropriate sensor for a given application or device it is important to consider several specifications, including the following:

1. **Size and Weight**: A wearable device should ideally be lightweight, so as not to increase users’ metabolic cost or fatigue, and be relatively unobtrusive and anthropomorphic. These requirements have previously been major factors limiting the use of these sensors for wearable devices, but advances in manufacturing and miniaturization has led to the development of some small, lightweight sensors.
2. **Range and Precision**: The desired range and precision of a sensor depends on its ultimate application and use. A depth range of 0.5–3 m is sufficient to allow for sensing any obstacles or terrain changes within several strides of a walking subject, while identifying objects in a seated task only requires a sensing range of 5 cm–1 m. Similarly, greater signal precision is required for sensing smaller objects, or for more sensitive tasks. Some sensors can adjust depth range to allow for sensing objects of different scales or at different distances, such as the Camboard Pico Flexx [21]. The desired field of view may also be very different, depending on the specific application and sensor position.
3. **Frame Rate**: Frame rate is an important specification to consider when selecting a sensor for use with a wearable robot. Sensors must be selected and operated at frame rates that are sufficient for motion estimation. However, there is a trade-off between data volume of high frame rate and computation time associated with processing more frames.
4. **Robustness**: To be practical for daily use, assistive devices must be robust to different environmental conditions, such as indoor or outdoor lighting conditions, or different terrains. Additionally, they must be robust to slight differences in position from day to day. Sensor-fusion approaches could be useful in cases where a given sensor is not robust to lighting changes or a specific environmental condition. Other approaches that could also be used to minimize this effect include using frequency modulation to compensate for ambient lighting [22].
5. **Portability**: Since the ultimate goal is to integrate teleceptive sensors into wearable assistive devices that can be used in activities of daily living in mobile scenarios, it is necessary to use lightweight sensors that work in an egocentric portable setup. This precludes the use of traditional gait or motion tracking systems that require fixed cameras and active or passive markers. Similarly, fixed-eye tracking systems are inappropriate for these purposes, despite their utility in other applications. Finally, though preliminary testing of any such sensors may be accomplished using a system tethered to a computer, ultimately any teleceptive sensor would need to be compatible with use in an embedded system.
6. **Cost**: The high cost of wearable robotic devices has been a major source of concern. For instance, until recently in the US, no insurance companies were willing to cover the cost of a powered exoskeleton, although select insurers are now willing to consider reimbursement for these devices on a case by case basis [6]. Decreased costs may enable substantially more widespread global acceptance and use of these devices, both in home and clinical settings. Thus, teleceptive sensors need to be low cost, so as not to increase overall device costs.

### 3.2. Sensing Modalities

A range of teleceptive sensing modalities exist at present. These include passive sensing technologies, which simply record from the environment, and active sensing technologies, which emit an energy source that is then sensed [11]. The only passive sensing technology that will be discussed here is a standard RGB camera, which senses visible light in the environment. The active teleceptive sensors that will be discussed either emit a form of electromagnetic radiation or sound, and then sense the reflected signal to understand the environmental conditions.

1. **RGB Cameras**: RGB cameras operate within the range of visible light, and typically either use pixels consisting of semiconductor charge-coupled devices (CCD) or complementary metal–oxide–semiconductors (CMOS) [23]. In each case, each pixel within an RGB camera consists of a sensor that transduces light into electrical signals. RGB camera sensors typically operate within a range of 5–60 Hz, while other teleceptive sensors may operate at frame rates of 1000 Hz or more. RGB cameras have become ubiquitous; they are found in smartphones, smartwatches, virtual and augmented reality headsets, and numerous other commercially available electronic devices. This makes them the first sensor modality that many researchers turn to when considering adding teleception. Some cameras are now small enough to be unobtrusively clipped onto a belt or pocket [24,25]; however, although these cameras are low cost, have good resolution, can be reasonably small, and are easily embedded, they have some disadvantages that limit their use with wearable devices.
  RGB cameras are highly affected by changes in lighting conditions in each space, such as the difference between indoor and outdoor lighting. Researchers have developed methods to compensate for differences in lighting, though these are still limited in that RGB cameras will not function in very dim or very bright light [26,27,28,29]. Additionally, although many other teleceptive sensors provide three-dimensional (3D) information, traditional RGB cameras flatten the space into two dimensions and provide no information about depth. Depth information can be obtained from individual cameras or stereo pairs of cameras (see below); however, this is less direct or precise than that provided by other sensor modalities. It is possible to use an RGB camera for wearable teleception without extracting three-dimensional data, but these approaches have limited ability to predict the distance to an object or a terrain change, which may be desired. Three main approaches have been considered to obtain depth information from RGB cameras:
  (a) *Monocular Cues*: Monocular cues, such as motion parallax, relative size, occultation, depth from motion, or perspective convergence can provide cues for distance to objects within the field of view [30]. However, some monocular cues, such as relative size, occultation, and perspective convergence are generally only useful for estimating relative depth between objects, rather than absolute depth. Motion parallax and depth from motion, on the other hand can provide rough estimates of absolute depth if the movement direction and velocity of the camera is known [30].
  (b) *Stereo Triangulation*: Most commonly, stereo triangulation has been used to estimate depth from two RGB cameras. This approach uses two cameras placed at a known distance from each other, which can be calibrated using a standard approach to estimate each camera’s focal length. Epipolar geometry defines the position of an observed pixel on the image planes of each camera and, if these image planes are aligned using a rectification transformation, the distance to the observed pixel can then be computed using the focal lengths of the cameras and the distance between them [31].
  (c) *Coded Aperture*: This approach uses an estimation of blur, which will increase with increasing distance in a properly focused camera. The aperture of the camera has multiple distinct openings to allow for a unique blurring pattern, and, if the aperture mask is known, it is then possible to estimate depth from a sharp image and use this information to deblur other images and estimate the depth of pixels within these images [32]. This method is still relatively new and has not been extensively implemented in teleceptive sensors, but it is a promising method for performing depth sensing using a single RGB camera.
  (d) *Dynamic Vision Sensors (DVS)*: These sensors were developed to mimic the human retina, in which signals are only produced if there is a visual change [33]. Using a DVS reduces processing requirements and is ideally suited for flow-based estimation and for high-speed recognition of changes in the field of view, without the need for a high-speed camera or computationally costly data processing or storage. These sensors do require at least a slight movement of the object or sensor for image production, but for wearable assistive devices this is likely not an issue.
2. **Laser/LED-Based Sensors**: Though less ubiquitous than RGB cameras, Laser- and LED- (Light Emitting Diode) based sensors have become increasingly prevalent in electronic devices, including smartphones, video game systems, drones, and autonomous vehicles. At times, these sensors are combined with RGB cameras and referred to as RGB-D sensors. However, unlike passive RGB sensors, these devices are active in that a signal is emitted and then the reflected signal is sensed, compensating for changes in lighting or for ambient infrared light using frequency modulation. These devices are ideal for estimating the distance from a sensor to surfaces in the environment, as follows:
  (a) *Slit scanners*: Slit scanners or Sheet-of-Light systems, emit a flat “sheet” of light, which is moved along an object or surface. By observing the deformed shape of the light with a camera, it is possible to estimate the shape and depth of the object or surface [34]. This approach requires knowledge of the position and orientation of both the light source and camera relative to the measured object. To measure the entire environment, you can move either the light source or the object, and both of these approaches have been used by 3D scanners such as Cyberware, ShapeGrabber, ModelMaker, and Minolta’s 3D scanner [35]. This technology does require a trade-off between field of view and depth resolution, though approaches have been taken to optimize these parameters.
  (b) *Structured light (SL)*: This teleceptive sensing approach was used in the first version of Microsoft Kinect. In this approach, a pattern of light (typically infrared, although other wavelengths are also used) is projected onto the environment. The pixels from the projected pattern are mapped back onto a camera to triangulate the depth of objects in space, similar to the way a stereo camera system operates [36]. To achieve accurate measurements from a structured light device, it is important to calibrate both the projector and the camera, preferably simultaneously (as proposed by Zhang, et al. in [37]).
  (c) *Time of Flight (ToF)*: These sensors compute depth by measuring the delay time from when a light pulse is emitted until its reflection is returned and sensed by photo-sensitive diodes. Using this specific, very small delay, and knowing the speed of light, it is trivial to accurately compute the distance to an object [38]. ToF sensors typically use infrared light, and, depending on the specific type of sensor, may provide anywhere from a single distance measurement to a 360-degree field of view. These sensors have become miniaturized in recent years, with recent integration into smartphones [39] and AR headsets [40].
    - *Laser rangefinder*: This is the simplest ToF sensor, which only requires a single photo-sensitive diode, as this type of sensor will only return a single depth measurement at a time [41]. These sensors can thus be used for distance estimation without significant computation; however, they do not provide as much environmental context as other teleceptive sensors discussed here. Additionally, this type of direct ToF sensor requires extremely high temporal sensitivity, to enable high precision depth measurements.
    - *Light Detection and Ranging (LiDAR)*: LiDAR has been used for several decades [42] and has become increasingly popular, particularly for autonomous vehicles, since the patent of Velodyne’s spinning LiDAR systems [43] in 2006. Using a spinning element as well as a large quantity of photo-sensitive diodes, it is possible to achieve a full 360-degree view. Although these sensors provide useful information, they are difficult to miniaturize and so are not ideal for wearable applications. An individual laser rangefinder can be operated at a much higher frequency and with lower computational cost than a full LiDAR sensor.
    - *Photonic Mixer Devices (PMD)*: PMDs modulate the outgoing light beam [44] using light-sensitive photogates and measure phase shift, rather than delay time, of the reflected light returned to each pixel to estimate the distance over the entire field of view. For each pixel, the system estimates the autocorrelation between the emitted frequency-modulated light and the returning reflected light, and uses this to estimate depth [22]. This technology reduces the need for delay precision to picoseconds, while still providing accurate depth sensing. Additionally, many of these devices use spectral filtering to reject ambient light sources and produce good depth sensing even in bright environments, including natural sunlight.
3. **Radar**: Similar to LiDAR, radar estimates the depth of positions in space by emitting a pulse and measuring the time delay (or frequency modulation) of the reflected pulse. However, the important distinction of radar is that rather than using pulses of visible light these sensors use low-frequency radio waves [45], leading to slower depth sensing. Challenges associated with using radar include diffraction of the beam as well as source separation in the case of multiple objects. Typically, radar has worse resolution and slower performance than LiDAR, though the sensing range is much further and is less affected by occlusions as radar can pass through thin objects. Radar has been proposed extensively for use in autonomous vehicles. Additionally, the use of radar close to the body has some potential safety concerns; however, low-powered systems do not pose substantial problems.
4. **Ultrasonic Sensing**: Unlike RGB- or Laser/LED-based systems that operate within the electromagnetic spectrum, ultrasonic sensors use high-frequency sound waves, above the audible range. Similar to ToF, ultrasonic sensors emit a pulse of sound and use the reflected sound, together with the known speed of sound propagation in the given medium (air, water, muscle, etc.), to estimate distance to the object. However, these sensors are affected by specular reflection, particularly with shiny or metallic surfaces [46].
5. **Sonar**: Sonar is similar to ultrasonic sensing, but uses very low-frequency, rather than high-frequency, sound waves. Typically, these sensors are used under water, though in theory they could be used in other media. However, as sonar is in the audible range, use of this sensor modality in an assistive device could be quite distracting. Unlike the radio waves used in radar, ultrasonic/sonar signals are unable to pass through thin barriers, such as windows [47].

### 3.3. Advances in Computing

In addition to the development of better sensors, several advances in computing have increased the feasibility of using teleception for assistive devices. First, a general increase in computing power and storage enables microcontrollers and smartphones to perform computing feats that only a few decades ago were beyond the scope of a supercomputer [48]. This in turn has led to the development of new algorithms and technologies to better use existing sensors [49]. An increased prevalence of sensors that can provide 3D data has led to the development of computer vision algorithms that work well in 3D, such as 3D segmentation, localization and mapping [20]. Increased use of deep learning techniques, such as Convolutional Neural Networks (CNNs) has also sparked rapid change within the computer vision field. CNNs have become popular because of their impressive classification performance, and the possibility of using deep learning approaches to select optimal features and parameters. Compelling results in the field of computer vision have thus yielded great promise for applying vision to other areas of study, which has likely facilitated an increased drive towards integrating these techniques into wearable assistive devices.

## 4. Teleception in Wearable Robotics

Researchers have tested the application of various teleceptive sensing technologies for use with assistive robots, such as wheelchairs [50,51], brain-computer interfaces [52,53], and canes for visually impaired individuals [54,55,56]. However, teleceptive sensing for wearable assistive robots is a very new area of research that only began within the last decade or so. In fact, several early proposals for teleceptive sensing in wearable assistive robotics used sensors that were fixed in the environment to remotely sense user behavior [57,58,59]. However, to be truly wearable, sensors must be portable, and algorithms must function appropriately in an egocentric perspective. This review is limited to wearable systems and will thus exclude applications using fixed cameras, motion capture systems, or gaze sensors.

Although this is a relatively new area of study, and most of the proposed approaches are still early in the validation and testing process, several have shown promise. The two areas that have been explored most thoroughly are the use of teleceptive sensors for grasp preshaping for upper-limb devices and intent prediction for lower-limb devices. Though the applications and methods vary, some characteristics are universal. Virtually all methods require the teleceptive sensors to be positioned outside of the individual’s clothing, either on the assistive device, or on a belt, armband, or headset, etc. Also, many of these approaches use inertial sensors to provide context for orientation of the teleceptive sensors relative to the environment.

### 4.1. Control/Intent Recognition for Wearable Devices

Correctly predicting the desired behavior of a wearable device is very important, because errors can result in unanticipated perturbations to the user and potentially lead to drops, spills, trips, or falls. Current control systems for powered assistive devices are generally multi-layered, with controllers at each layer performing different tasks.

High-level controllers define overall goals, such as the intended locomotion or manipulation activity. For example, in the case of a powered leg prosthesis, the high-level control system may use machine learning to predict the desired locomotion mode (i.e., level-ground walking, stair or ramp ascent or descent) based on kinematic and kinetic sensors in the prosthesis and electromyographic (EMG) signals from the residual limb muscles. Extensive work has been done to eliminate errors in high-level activity prediction [60], as an error at this level will propagate downwards through control layers, resulting in incorrect joint and actuator behavior. Neural signals, kinetics, and kinematics have been proposed for high-level activity prediction; however, recently several researchers have proposed the addition of teleceptive sensing (these will be listed in chronological order in the following sections).

Once the desired activity is predicted, mid-level controllers define the output of each joint. Generally, these consist of state machines that model the individual trajectories or speed of a given locomotion or manipulation mode. For example, a mid-level controller for walking could model the velocity, position, torque or impedance of the limb joints based on able-bodied locomotion. Recently, some researchers have proposed the use of teleception for mid-level control of joint behavior, such as in the endpoint control of an arm prosthesis [61,62] or the orientation of the foot during ambulation [63,64].

The joint-specific output of a mid-level controller feeds into the low-level controller, which controls actuation. Low-level controllers typically use a PID (proportional-integral-derivative) controller, or a similar control architecture, to modulate position, torque, or velocity of a given joint and to ensure that the correct motor commands are generated. This control level is, at present, the least suitable for addition of teleceptive sensing.

### 4.2. Upper-Limb Prostheses and Exoskeletons

In standard two-site myoelectric control, upper-limb prostheses (and orthoses [65]) are controlled using EMG signals to independently control each joint or degree of freedom (DoF). This can provide users with proportional and direct control of each desired joint, but coordinating control between multiple DoFs can be quite challenging. The burden of controlling multiple DoFs becomes impractical, for example, for individuals with an amputation at the shoulder level who must coordinate multiple joints at the shoulder, elbow, wrist, and hand. Performing all these computations simultaneously is extremely challenging and places the primary control burden on the user, making the use of such prostheses arduous and slow. To reduce the user’s control burden, researchers have proposed high-level controllers that predict the desired grasp type and endpoint location and fully or semi-autonomous mid-level controllers to adjust behavior of individual joints. Several research groups have considered the use of teleception for high-level grasp prediction and endpoint control, as detailed in the following sections and summarized in Table 1.

#### 4.2.1. Grasp Prediction

One of the most widely explored applications for teleceptive sensing in wearable assistive robots has been object detection for grasp prediction. Recent research towards this end has primarily used either a sensor-fusion or machine learning approach.

a. Sensor-Fusion Approaches

A “cognitive vision” system based on an in-hand webcam and an ultrasonic rangefinder designed for grasp preshaping was first presented by Došen and Popovic in 2010 [66]. This system was tested and described in several publications as it evolved over the next several years. In the original system, the webcam was used to segment the object closest to the center of the image and the actual size of the object was estimated using the distance to the object provided by the ultrasonic sensor and the length of the object in pixels. Once the object was segmented and its size estimated, the desired grasp type was identified from one of four possible grasps, based on the object height and thickness, and the desired wrist orientation was estimated based on the angle of the long axis from horizontal. Proof-of-concept demonstration of the system showed size estimation errors of up to 25%.

Shortly thereafter, an experimental evaluation of the vision system used together with dual-site EMG to control a prosthetic hand mounted on a splint was presented (see [67]). In this implementation, a laser pointer was also included to better identify the desired object prior to segmentation. Once the desired grasp type was identified as previously described, the hand was preshaped prior to voluntary initiation of hand closing, based on a flexion EMG signal. An extension EMG signal would then indicate a desire to release the object. Subjects tested system performance in grasping trials. Overall, the task was accomplished 90% of the time, with the correct grasp type and size being estimated 84% of the time. Time to grasp an object, move it to a target location and position it above the target was also computed, with a completion time of 19.4 s in the first testing block, and 10.4 s in the sixth and final block, indicating that subjects learned to use the system, although hand preshaping was slow and only 84% accurate.

This control system was then modified by Markovic, et al. with the integration of augmented reality (AR) glasses with stereo RGB cameras and two EMG channels to control the prosthesis [68]. Stereo cameras in the AR glasses were used to obtain a depth image of the field of view, to which a geometrical model (box, cylinder, sphere or line) was fit, for estimation of desired grasp type and hand aperture. Subjects were also provided with visual feedback about the predicted object and aperture on stereo displays within the AR glasses. Based on this visual feedback, users could use EMG to trigger opening of the hand and adjust the hand aperture. Additionally, once the hand was in the vicinity of the desired object, subjects could use EMG to command hand closure or reopening, if desired. Testing of this control system by 13 able-bodied subjects showed that a semi-autonomous system that included AR feedback yielded higher task performance (81%) and grasp success rate (94–99%) than either a semi-autonomous system without AR feedback or a fully autonomous system.

An extension of this work was presented in [69]. In this case, an RGB-D camera placed on a wearable glasses-based headset replaced the stereo camera. In addition to hand preshaping, the teleceptive sensor was used for controlling wrist rotation, and an IMU was positioned on the wrist to sense wrist orientation. The user could trigger automatic hand preshaping and wrist rotation using a short burst of EMG. Subjects could then use EMG to adjust individual DoFs (and use co-contraction pulses to switch between DoFs). This system was tested online with ten able-bodied subjects and one amputee subject. Subjects were instructed to grasp objects of differing size and shape, lift and transport them to a container, release them, and then return their prosthesis to a starting position. The semi-autonomous system was compared to systems where individuals had direct, sequential control of 1, 2, or 3 DoFs using EMG alone. The time to grasp was lower for the semi-autonomous system than a 3 DoF direct control system, but slower than the 1 or 2 DoF direct control systems. However, the semi-autonomous system resulted in fewer compensatory movements, such as shoulder abduction or external rotation, than the 1 or 2 DoF systems. The integration of teleceptive sensing and EMG in a controller for a prosthetic hand presented in this series of papers was extremely promising, demonstrating proof-of-concept, a reduced time to achieve the grasp, a reduction in compensatory movements, and improved performance relative to fully autonomous or fully direct control systems.

Other research groups have considered different approaches for automatic teleception-based grasp selection systems. In [70], an RGB webcam attached to a hand, together with mechanomyography (MMG) on the contralateral forearm were used to operate a Bebionic hand [1]. RGB images were segmented into regions of interest (ROIs), and a Gaussian filter was used to smooth these images and detect edges, which were then used to identify objects within a given size range, achieving an average recognition rate of 84%. Once objects were identified, the desired grasp type was identified as either a power or a pinch grasp, and filtered MMG was used to drive the opening and closing of the hand within the desired grasp. Robustness of this prediction to cluttered environments and other objects was not considered extensively.

Finally, Giordaniello et al. considered a different approach to grasp prediction, using a fusion of EMG, gaze, and RGB images [71]. A wearable headset provided gaze information and RGB images, which were used to estimate the desired object in a busy environment, as well as providing an estimate of where and how each object was to be grasped. Additionally, EMG signals from 14 sensors on the forearm were recorded, and Cyberglove data were recorded to give an estimate of the joint angles during each grasp. Predictions based on EMG data alone yielded relatively low accuracy (75% after data relabeling), signaling the need for improvements, potentially by combining gaze and vision, though this was not tested explicitly.

b. Machine Learning Approaches

In the last few years, deep learning has led to rapid developments within the field of computer vision, and several research groups have published early results of using an RGB camera and a trained CNN to predict desired grasp type. In one such approach, DeGol et al. [72] used RGB images from published datasets to train a CNN to predict one of five grasps. Images were then captured with an in-hand RGB camera to test the classifier, resulting in a grasp-type prediction accuracy of 93.2%. Ten real-time tests were then completed in which classifier was used to actuate the hand in real time into the desired grasp type, though these results were not quantified.

Similarly, Tang et al. [73] used a DVS [74] attached to a multi-joint prosthetic hand (iLimb [3]) to estimate grasp type and orientation of objects relative to the hand. A CNN was trained to predict desired grasp type, and symmetry was used to identify the central axes of desired objects, which were then used to determine object orientation. The CNN classified with 98% accuracy, and the correct orientation was identified within 10 degrees in 96% of cases. This result is promising, although testing online or in cluttered environments has not yet been done.

Meanwhile, Ghazaei et al. [75] presented a different deep learning-driven approach for grasp prediction, with preliminary testing in amputees. Images from the ALOI database plus supplemental photographs (made available as the Newcastle Grasp Library) were processed to train a CNN. The predictive system was implemented in real time, after comparing different CNN architectures and cross-validation approaches. A CNN that yielded 70% offline performance on average was tested by two transradial amputees. The subjects were instrumented with forearm EMG electrodes as well as a single webcam attached to the dorsal surface of an iLimb prosthetic hand. Subjects flexed muscles for a short period to trigger the camera to take a picture, then, if satisfied with the image, completed pick-and-place tasks with a variety of objects. In the first few experimental blocks they were provided with visual feedback of the segmented object and their EMG signals, but in later blocks no visual feedback was provided. Accuracy was approximately 79% in the last (and most accurate) block, which also had 88% task completion. This study showed that subjects could learn to use this sensor-fusion driven system for completion of pick-and-place tasks, even when accuracy was less than 80%.

Likewise, Bu et al. used a CNN to classify RGB images of 22 different objects, each of which required a defined specific grasp type [76]. Dual-site EMG amplitudes were then used to drive the prosthesis forward or backward (or in this case, hand open or close), in the planned grasp. A single able-bodied user tested the EMG system, with sensors placed on the contralateral arm, while the individual held the prosthesis in place to perform grasping. The forward or backward motion of the hand were not quantified, though performance was reported as being good. Although the image classifier had 90% accuracy, these results are limited because the vision sensor was not actually placed on the hand or elsewhere on the body; the classifier was instead tested using static images of the different objects.

Taverne et al. [77] presented a deep learning predictive system that used depth data rather than simple RGB images. The goal of this work was to develop a low-latency system that did not require an “aiming phase”, unlike several of the previously described approaches (e.g., [69,75]). An RGB-D sensor was worn on an armband during reach-to-grasp motions, and deep learning models, based on per-frame ResNets and a Long-Short-Term-Memory (LSTM) artificial recurrent neural network, were trained using “hard” and “baseline” Handcam datasets, to predict between six grasp types and a no-motion class. The baseline dataset had good classification accuracy of up to 96%, but including new objects or locations into the hard dataset decreased performance to 89% and 76%, respectively. However, a major benefit of this approach was that the subject completed grasping in a natural manner, without needing to aim or pause during the process.

#### 4.2.2. Endpoint Control

As an alternative to estimating the desired grasp type, a few research groups have proposed the use of teleceptive sensing for endpoint control of assistive devices for the upper limb. The purpose of an endpoint controller for a prosthesis or exoskeleton is to reduce the control burden, i.e., the need to adjust each joint individually, on the user. Instead, the position of the endpoint is controlled (or predicted), and then the trajectory of all joints are mathematically computed based on this desired endpoint. Here we present a survey of publications that detail the development or testing of an endpoint controller for wearable robotics based, at least partially, on teleceptive sensing.

Martin et al. [78] describe a system to predict grasp type and object location for a prosthetic arm using EMG and RGB-D data from a helmet-mounted Microsoft Kinect v1. This system uses color-based blob detection to estimate the desired object, and EMG is used to toggle between segmented objects. Once the desired object is identified, the system locates the object in 3D space and computes the necessary arm movements to achieve this endpoint. Images from preliminary tests of this autonomous system are shown, but its performance is not quantified in this work. In the proposed system, motion estimation based on IMUs would ultimately be integrated into the final prediction to improve localization of the prosthesis.

Madusanka et al. [61] described a reach-to-grasp planning system based on vision and EMG. In this system, elbow flexion and extension are driven by an EMG module that is fused with a visual servoing module, which aligns the palm with the desired object. The elbow joint is controlled using a combination of the EMG and visual servoing modules. The EMG module was tested using a 5 DoF prosthesis, and the visual servoing and fused systems were tested using a simulated environment with a 5 DoF virtual prosthesis that was designed to match the kinematic chain of the actual prosthesis. The visual servoing module moved the virtual prosthesis to try to reduce the orientation error of the grasped object. Elbow flexion/extension was jointly controlled by the user and the visual servoing module, with an RMSE of 10.8 degrees. Though the error was dominated by the EMG, the visual servoing did improve performance, particularly for faster movements.

Finally, Krausz et al. [62] also proposed an endpoint control architecture for performing pick-and place tasks, consisting of reaching, grasping, and repositioning phases. Seven able-bodied subjects were instrumented with EMG sensors on 14 muscles in the arm and a wearable glasses-based gaze tracker. As head-mounted gaze tracking is prone to misconstrual due to head-movements, a system was developed to filter the tracked gaze using knowledge of saccades and gaze fixations, and computer-vision-based movement estimates between consecutive frames were recorded by a head-mounted RGB camera. Regression-based estimates of the endpoint position in the x- and y-directions were produced using EMG and gaze. A Kalman Filter [79] sensor-fusion algorithm based on the natural biomechanical coordination between movement, gaze, and EMG was used to fuse EMG and gaze-based endpoint position estimates throughout each trial and at the end of each repositioning movement. Finally, a “simulated amputation” case was considered, in which only shoulder muscle EMG was used for endpoint predictions. The results showed that the addition of gaze could compensate for removing EMG signals from the upper arm and forearm, and the overall positional RMSE was <7 cm. Ultimately, the authors proposed an inverse kinematics mid-level controller to drive individual joints upon endpoint identification, and real-time testing of this fused endpoint predictor in amputees.

Substantial work has been done to consider the addition of teleceptive sensing to the prediction and control of hand preshaping for upper-limb prostheses, and this approach seems to be promising, although none of these systems have yet been clinically implemented. Additional testing of these systems in realistic activities of daily living, cluttered environments, and with amputees will help provide greater insight into the ideal system for translation out of the lab. In the future, a general metric and testing scenario might also prove useful to allow better comparison between different systems, algorithms, and published data. Recently Saudabayev, et al. [80] published a freely available dataset of 13 able-bodied subjects completing activities of daily living while wearing 5 IMUs on the chest, arms, and forearms; an RGB-D sensor on the dominant forearm; and an RGB camera on the head. This dataset, which included annotations of the desired grasp type, could eventually be useful for comparing different sensor-fusion or grasp-prediction algorithms.

### 4.3. Lower-Limb Prostheses and Exoskeletons

State-of-the-art lower-limb prostheses and exoskeletons use a hierarchical structure in which a high-level controller estimates the overall desired locomotion mode at each identified gait event (heel contact or toe off), a mid-level controller estimates the desired behavior of the limb during the specific locomotion mode and gait phase based on biomechanical models of able-bodied walking, and a low-level controller modulates motor commands to achieve the desired behavior [81]. Within this hierarchical structure, several potential venues exist to improve performance by incorporating teleceptive sensing. Preliminary testing of a few applications within the high- and mid-level controllers in the gait segmentation, terrain detection/intent recognition, and mode-specific control processes are detailed below. Table 2 presents a summary of the publications that discuss teleception for lower-limb assistive devices.

#### 4.3.1. Gait Event Detection

Forward prediction of the desired locomotion mode for a powered prosthesis or orthosis occurs for each step at identified gait events, such as heel contact, toe off, midstance, or midswing. Gait events such as these can also provide context for the biomechanics of the individual walking, such as symmetry, dual support time, and more. These gait events have typically been detected using load, contact, or motion sensors in the wearable device [82]. This approach is direct and reliable; however, not all wearable devices have these sensors. Additionally, prosthesis users typically do not have additional sensors placed on the contralateral limb, thus coordination between the limbs is not feasible. Several non-wearable methods, including using sensorized floormats [83], motion capture [84], or camera sensing [85] can be used effectively for gait analysis, but as they are not portable, using them for everyday control of a wearable device is not feasible. The use of a wearable teleceptive sensor has recently been proposed for this purpose.

An algorithm to predict gait events based on unilateral, collocated thigh-mounted ToF depth and IMU sensors was presented in [86]. This work was intended to be device-agnostic, with a simulated “prosthesis” assumed to be worn on the right side, and thus double support was predicted only using sensors mounted on the right thigh. An SVM was trained to predict gait events based on the thigh IMU. The contralateral leg was visible within the depth sensor field of view, as was the environment, and features from the motion of these objects in space were used to train a predictor, based on a novel template-matching approach. The predictions from each sensor modality were then linearly combined, and results from walking trials with a single able-bodied subject showed that fusion of data from a single depth sensor and an IMU were able to approach the ground truth, with gait event offsets of 6 ms, and F1 scores of 92.5%. The authors speculate that the benefit of adding depth sensing would be greater during walking by subjects with gait abnormalities or asymmetries, or for coordination between two devices.

#### 4.3.2. Forward Prediction

Once a gait event is identified, the locomotion activity of the following step needs to be classified. While it is theoretically possible to use a key fob or an irregular movement to signal the desired activity, performing this on every single step would be rather tiresome and slow. For some steps, such as in the middle of going down stairs, it may also be dangerous [60], particularly for individuals who are already somewhat unstable. Thus, forward prediction of the desired locomotion activity is necessary. A mid-level, gait-phase specific, activity-based controller can then be used to control the trajectory, stiffness, or other parameters of the robotic device. Previous work has proposed the use of machine learning-based forward predictors based on EMG, kinetics, and kinematics to estimate the desired locomotion activity. More recently, several research groups have explored using teleceptive sensing of terrain for use to improve forward prediction of desired locomotion activity.

a. Geometric Segmentation Methods

In one of the earliest attempts to use teleceptive sensing for improved performance of a powered lower-limb prosthesis or exoskeleton, Zhang et al. [87] proposed the use of a laser distance sensor, which would provide a single distance measurement that could be used to predict the terrain height based on the known height of the sensor, an integrated IMU signal, and knowledge of typical terrain geometries. The IMU was used to distinguish between standing still and movement or turning, and the terrain recognition was produced using the laser-determined distance and a rule-based algorithm. This system was evaluated using a timing threshold, whereby a terrain transition needed to be correctly identified within a critical timeframe, otherwise it would be considered erroneous. This system achieved an average overall accuracy of 98% with a terrain prediction time of 2.3 s.

Ultimately the goal of this work was to use these predictions to adjust the prior probabilities of the intent recognition system for a prosthesis. Implementation and preliminary testing of this system, described in [88], was based on similar methods and using the same sensor modalities as described in [87], with an IMU and a laser rangefinder placed laterally at the waist. EMG signals from the lower limb and ground reaction forces and torque signals from a 6 DoF load cell were used to train an LDA locomotion activity classifier. The a priori probabilities of each activity were then adjusted using coarse and refined terrain recognition modules. The prior probabilities were defined differently for steady-state and transition steps. Six able-bodied and one amputee subject were recruited to test the integrated system. First the terrain recognition was tested in able-bodied subjects ambulating in a laboratory-based circuit comprising level-ground walking, stairs, and a ramp. The terrain recognition accuracy was 99% for the refined output and 98.6% for the coarse output, and the response time was positive, indicating that the terrain was recognized prior to critical transitions. Next, two able-bodied subjects and a single amputee walked with a powered knee over different terrains, with a researcher triggering activity transitions remotely. Desired activity was classified with and without teleceptive sensor information, and classification accuracy was found to be improved, with an average of 95% accuracy when incorporating either the coarse or refined terrain recognition data. Finally, the amputee walked in an unstructured environment with an online decision system, and the error count was reduced from 57 without any terrain information to 21 using the coarse output, and to 11 when using the refined output. These results were promising, although additional clinical evaluation is needed.

In a similar approach to that presented in [87], Carvalho, et al. [89] used a laser distance sensor to estimate locomotion transitions. A subject-specific dynamic calibration was used to determine the thresholds for a three-layer decision tree that was designed to identify level ground, ramps, or stairs. This decision tree was used to heuristically classify sliding windows as a given locomotion activity. The system was validated with 10 able-bodied subjects walking on a series of terrains, as an assessor labeled each of 400 total locomotion transitions. The average prediction accuracy of 92.2%, may have been affected by poor laser distance estimates due to reflective surfaces. The average prediction time was 1.99 s prior to the actual locomotion transition, which highlights the possibility of producing forward predictions significantly in advance using teleception. Ultimately, the goal of this work would be to incorporate the locomotion prediction into an overall forward prediction system as a prior probability, as described in [88].

The first attempt at using 3D data for gait activity prediction for powered prostheses (as a means of extracting additional information from the environment than that provided by a single laser rangefinder) was presented in [90]. This work built upon previous 2D activity prediction proposed in [91]. In this case, a Microsoft Kinect v1 structured light RGB-D sensor was used. Because a standard method for locating stairs from 3D point-cloud data was lacking, and the methods proposed were not designed to produce estimates of user intent, a novel segmentation algorithm was proposed to locate stairs in the environment. This algorithm also produced a series of secondary features that could be used to estimate user intent, including distance from the user to the stairs, the angle of approach, step depth, step height, and step count. The vision algorithm was tested in several offline conditions, including at different positions and with different staircases, and results showed that the computed distance to stairs correlated well with the measured distance, the mean angle of approach was estimated within 1° of the measured angle, and general distance errors were on the order of 5 cm. Finally, an online walking test was performed with a single able-bodied subject walking on level ground and stairs, with forward predictions of level-ground walking or stair ascent produced in real time at 5.15 frames per second (fps). An accurate prediction was obtained in 98.8% of tested frames, and 100% of steps taken were accurately predicted. The algorithm has since been expanded to include ramp walking and stair descent and to use a small portable depth sensor [21] with an attached IMU to estimate the depth sensor orientation [92].

This early work led to a proliferation of publications describing forward prediction approaches for prosthetic legs or exoskeletons using teleceptive sensing, primarily using LED/Laser depth sensors, though other approaches were also used. For instance, Zhao, et al. [93] describe an algorithm designed specifically to identify ascending stairs in the environment using a Kinect v2 and an IMU to obtain geometric parameters. First horizontal and vertical planes are identified using a normal-vector estimation procedure. Then, by considering the intersection of horizontal and vertical planes, the algorithm identifies stairs, and computes step count, horizontal and vertical distance to a step, step height, and step depth. This approach is quite similar to that described in [90], although in this work, planar surfaces were identified using RANSAC [94]. Two datasets were collected with a subject walking on different staircases while the Kinect was strapped to their waist. Overall processing time for the two datasets was 18 ms per data frame. The authors propose using this algorithm for forward prediction in a lower-limb exoskeleton.

A slightly different approach is presented in [95], in which a depth sensor was used to identify terrain and locomotion activity, without an IMU. Stair edges were identified from depth point clouds using a Hough Line transform, and an edge was identified as belonging to a stair using the change in depth values and the angular change of the surfaces on either side of the edge. Once stair edges were detected, a finite state machine was designed to identify the specific locomotion activity, as either standing still, level-ground walking, stair ascent, or stair descent. The system, tested in 9 able-bodied subjects walking around a circuit of different terrains, predicted the desired steady-state activity with an accuracy of 100% and transition events with an accuracy of 82%. In the future, the authors plan to validate this approach in real time with an exoskeleton.

Unlike previously discussed teleceptive sensing systems, Kleiner et al. [96] proposed the use of radar rather than light or laser sources for forward prediction, to reduce the effect of clothing or occlusions on performance. A Frequency-Modulated Continuous Wave (FMCW) radar sensor was attached to a BiOM ankle prosthesis [2], along with an IMU to identify gait events, and a motion capture system and sensorized insole were used to obtain the ground truth locomotion activity for comparison with the radar predictions. Stair presence and height could then be found using the radar signal, which requires processing, as radar provides a measure of the reflection being returned to the scanner rather than the actual distance (as provided by a ToF sensor), and the signal is a function of the radial distance to an object. This radius varies based on size, geometry, surface material, and more, and thus to obtain the exact height of a reflected stair, some assumptions must be made. Ultimately an approximate value for heights of objects in the environment can be computed, although an understanding of how different terrains affect the reflected radar scan, and how well this can be used for forward prediction, requires additional study.

b. Machine Learning Approaches

Varol et al. tested an RGB-D sensor worn on the shank and oriented towards the ground [97]. A single able-bodied subject walked in natural environments with a variety of lighting conditions, and five activity modes were labeled. Depth differences between two frames were used to provide context about the motion of the environment relative to the user. Statistical features were then extracted from ROIs at 4 different sizes and used to train an SVM classifier with 5-fold cross-validation. The SVM model with the highest accuracy was used for prediction, and a majority voting filter was applied to improve prediction stability. Preliminary results demonstrated good performance with 99% activity accuracy, and a single transition prediction error.

The system presented in [97] was modified slightly and validated with an additional 12 subjects in [98]. As in [97], subjects walked over several different terrains while wearing an RGB-D camera, attached at an angle, on the right shank and an RGB camera strapped to the chest to label frames. Statistical features were standardized prior to dimensionality reduction, SVM training, and majority voting. Several dimensionality reduction and SVM strategies were compared, and the best accuracy was produced using a cubic SVM kernel with no dimensionality reduction. However, this also resulted in the highest computation time. This strategy was selected to optimize performance, but may have introduced lag into the system. An average steady-state error rate of 0.75% and transition error rate of 6% were obtained, with an average recognition delay of 0.33 s. Training the SVM classifier using 8 subjects, and then testing using the remaining 4 subjects resulted in an average classification accuracy of 94.5%. However, 10% of correctly classified transitions had prediction delays exceeding 0.5 s. Though these preliminary results demonstrated the potential generalizability of teleceptive sensing for forward prediction, a higher prediction accuracy and lower prediction delay, to avoid causing trips or falls, would be required if integrated into an assistive robot.

Since the introduction of deep learning, particularly CNNs, into the computer vision field, several groups have presented approaches to solving the forward prediction problem using a CNN trained on teleceptive sensor data. For example, Laschowski, et al. [99] presented a CNN-driven approach in which an RGB camera was attached to the chest while the subject walked in a variety of environments and lighting conditions, and images were captured at 60 fps. Millions of images were obtained during 10 h of walking, and a fraction of the images acquired during the experiment were labeled by an experimenter as level-ground walking, stair ascent or stair descent. A 10-layer CNN was then trained with spatial pooling and stride-based pooling, and 5-fold cross-validation was implemented. The overall classification accuracy was 94.9%, with descending stairs achieving the poorest accuracy of 87.3%, though this may be due to an imbalance of images for each class, and partially due to false negatives.

Similarly, Novo Torres et al. [100] used an obstacle detector based on a CNN, created using RGB camera images acquired during 7 h of walking. Images were labeled as either containing an obstacle or not, and split into validation and training sets. Training accuracy for the CNN was 98.7% and the validation accuracy was 95%. However, images from an unseen trial produced an overall obstacle prediction accuracy of 89%. In the future, the authors plan to include other sensors, such as an IMU, and to implement this system on a prosthetic leg.

Khademi, et al. [101] also presented results from using a CNN with RGB images. In their implementation, an IMU on the thigh was used to trigger a camera attached to the waist. Transfer learning using a pre-trained VGG-16 model [102] was used to train a CNN in a computationally efficient way, and the training and validation data sets yielded >99% accuracy. The CNN was trained to predict level-ground walking, stair ascent, or stair descent, and the prediction accuracies were on average 99% for steady-state locomotion and 90% for transition steps. Including prediction history improved transition accuracy by taking into account the number of steps in a staircase. Once this information was included, the overall accuracy for all steps was 99%. Ultimately the authors intend to use depth images rather than RGB images alone to optimize performance.

Zhang et al. [103] presented results of a CNN-driven terrain detector that used depth-based point clouds rather than RGB images alone. A depth sensor and an IMU were strapped to the thigh during indoor and outdoor walking trials with 6 able-bodied subjects and 3 amputees. In this approach, 3D point clouds were dimensionally reduced so that only the x-z (or side view) projection was extracted and saved as a binary image, from which features could be extracted for training or testing with a deep CNN. From this binary image, RANSAC [94] was used to fit lines to the edges of the terrain. Simulated binary images were used to test the terrain recognition and achieved 100% accuracy. Walking trials in different environments were then used to test the overall system performance, and achieved 95% accuracy, with an average time of 3 ms to process each frame, although this was not tested on an embedded system. Additionally, environmental features, including the height and width of stairs and the slopes of ramps, were estimated for future integration into an activity-specific mid-level controller.

The results of CNN-based approaches are promising; however, at present the implementation of a deep learning classifier is computationally costly and has yet to be tested with an embedded system. It may be some time until it is realistic to use deep learning in an embedded real-time forward prediction system for a wearable robotic device.

Krausz et al. [104] used a machine learning approach to compare teleceptive ToF depth sensing to proximate sensing using IMUs, goniometers, and EMG for prediction of desired locomotion activity. Several depth features were extracted from point clouds acquired during able-bodied walking over a range of terrains. The features were normalized and dimensionally reduced using LDA, and then the variability, repeatability, and separability of these features were assessed, relative to IMU, goniometer, and EMG features typically used for activity forward prediction for powered lower-limb prostheses. The depth-based features had low intra-subject and intra-activity variability, between activity separability, and between subject repeatability, thus by combining depth features with the other proximate feature types, overall repeatability between subjects and separability between activities was improved. These results may allow development of a controller that is generalizable across different subjects and different activities. In an individual with a transfemoral amputation walking on a powered prosthetic leg, Krausz et al. (under review) [105] found that this offline controller, with a fusion of teleceptive and proximate sensor modalities, was better than a controller that did not include teleception.

#### 4.3.3. Activity-Specific Control

Once the desired locomotion activity for the next step is predicted, a wearable assistive device requires a mid-level, activity-specific controller. Such controllers are designed to mimic the impedance of able-bodied walking [4] or to follow natural trajectories or parameters of gait [106,107] for a specific locomotion activity. However, minimal research has been performed to use teleception for modulating the mid-level control of a wearable device, though we expect that this will be a promising area of research.

Preliminary results of using a teleceptive-driven control system for an assistive device was described by Scandaroli and colleagues [63]. This paper presented an approach to estimating the foot orientation for a prosthesis using a gyroscope and IR distance sensors. The prosthetic foot had two gyroscopes and four IR distance sensors, which provided inputs to an Extended Kalman Filter that fused sensor data for an estimate of the foot orientation. This estimate was then compared to ground truth based on a potentiometer with a known initial position, and found to be quite good, with directional errors as low as 0.01%. This information could potentially be used to control a prosthetic leg; however, exactly how the integrated system would operate if, for example, a shoe is worn, is unclear.

Several papers have discussed the possibility of using parameters extracted during forward prediction in the control architecture of wearable devices. For instance, [90,93,108] presented and validated measures of stair height and depth that could potentially be used to modulate toe off or power for walking on different terrains. However, as this is still an emerging area of research, identifying the ideal use of teleception for modulating the control of an assistive wearable device still requires significant study.

Finally, Liu et al. recently published a study evaluating an obstacle detection system, based on an RGB-D sensor [64]. In this paper, an integrated control system for a powered exoskeleton was tested. First, point clouds would have the ground filtered using RANSAC [94]. Then remaining objects would be considered to be obstacles. The system would then use the obstacle height and width to model the gait pattern required to either stop before encountering an obstacle or to step over an obstacle. Based on the proximity of the obstacle, a rule-based decision-making algorithm would be used to decide whether to continue walking or to stop. This decision would then automatically initiate the termination or obstacle-clearing step. The control system was tested on a powered exoskeleton by 3 able-bodied subjects, and both the obstacle recognition and the decision-making behaviors were evaluated, with few failures reported. However, how the autonomous behavior of the obstacle detection system would affect real-time behavior of subjects walking in the device was not determined.

## 5. Discussion

The use of teleceptive sensing for improved performance and behavior of wearable assistive robotics is a new and promising area of research. Here we present a discussion of sensing technologies and the necessary conditions for effective use of a teleceptive sensors, together with a survey of the research that has been completed to date towards implementation of these technologies into wearable assistive robotic devices. In particular, a growing number of researchers have been investigating the use of various teleceptive sensing technologies for grasp preshaping and endpoint control for powered upper-limb devices; and gait segmentation, forward prediction, and activity control for lower-limb devices. The two areas that have been explored most thoroughly are object detection for grasp prediction or preshaping, and terrain detection for locomotion mode forward prediction.

For grasp preshaping, the most common approach has been the use of an RGB camera, either in the hand or on a glasses-style headset, often combined with EMG sensors or IMUs to provide a sensor-fusion-driven grasp prediction. Both traditional, geometric-driven object segmentation approaches and CNN-based deep learning approaches have been used to identify the desired object and grasp. In general, the performance of these systems has been good, with accuracies in the 75–96% range, which is comparable to state-of-the-art EMG-based approaches. However, concerns remain about timing and lag of these approaches. Additionally, it is unclear how well these devices will operate in occluded or real-world environments, particularly due to the variability of environmental conditions that may be present during testing. Several studies have demonstrated integration of a grasp preshaping system into control of an upper-limb device, and some have included individuals with biomechanical pathologies (see [69,75]); however, more work is required to validate these systems. Fewer groups have attempted to use vision for endpoint control of a prosthetic or orthotic arm, and no such system is as yet fully integrated and validated. However, preliminary results show the promise of this approach, and we expect that it will be considered more extensively in the future.

For lower-limb assistive devices, the most common approach is mounting depth-based RGB-D or IR sensors either on a belt, at the waist, or on the shank, along with IMUs to provide rotational context to resolve the point-cloud sensor data into global coordinates. Most commonly these systems have focused on performing terrain detection to estimate the desired locomotion mode activity at segmented gait events. Though several studies have presented preliminary results for forward prediction, few have presented integration with an actual device, with the exception of [88,96,105], and to our knowledge only [88,89,105] have performed testing in amputees or in other individuals with gait impairments. However, results from various studies with able-bodied subjects and mostly offline testing have shown great promise, with an 82–99% activity prediction accuracy. As with upper-limb applications, additional testing and validation is required prior to the translation of these technologies out of the research laboratory.

### 5.1. Barriers to Clinical Translation

Though the addition of teleceptive sensors to wearable assistive robots has great potential for improving control of these devices, numerous obstacles must be overcome before these systems can be effectively translated to the clinic or used in daily life. First, streamlined, tetherless, robust systems in sturdy packaging must be developed to prevent hardware issues and allow subjects to use their assistive device daily. In addition, it is essential that teleceptive systems are tested and characterized to understand how they perform in different lighting conditions, in occluded or busy environments, and across a wide range of natural human movements and behaviors. For this reason, we recommend use of a sensor-fusion approach in which inertial sensors, EMG sensors, or other patient-driven sensors are incorporated together with teleceptive sensing to obtain a more complete information about the desired behavior. Additionally, using multiple sensors reduces the risk of a system failure due to a malfunctioning sensor.

In general, a greater understanding and additional testing of the algorithms and sensors being used will be necessary before any of the proposed approaches is suitable for daily use. For instance, it is not yet clear how less prescribed movements seen outside of a gait laboratory would affect an intent recognition system based on teleceptive sensing. Addressing this issue will require extensive testing with numerous users, in different environments, and over multiple days before a take-home clinical trial. Most of the preliminary studies described in this review were performed offline, or predictions were made online without being integrated with a real-time wearable device, which is necessary before use outside the laboratory.

Another barrier that must be addressed is ensuring that individuals are confident in the behavior of the device and control system. It has been shown [109] that one reason assistive devices are abandoned is lack of user confidence, often associated with fear of falls, or poor controllability. This may be of particular concern for more autonomous systems. Therefore, it is important to develop computationally robust and responsive systems that do not induce undesirable or unpredictable lag. Performance needs to be stable, with low error rates; require few compensatory movements; and result in few perturbations to the user across days. Additionally, higher risk activities, such as stair descent, must achieve even lower error rates to ensure that users feel safe. This important consideration is potentially also an area where teleceptive sensing may reduce misclassifications.

### 5.2. Potential Directions of Research

Several exciting pathways exist to include teleceptive sensing into the control architecture of powered wearable assistive devices. In addition to expanding on prior work on their integration into wearable device control systems and validation in online, real-world settings with relevant target populations, several open questions must be addressed. First, although it is clear that teleceptive sensor modalities can provide environmental context that has previously been missing, the importance of the content provided is unclear. For instance, little work has been done to compare different teleceptive sensor modalities or teleceptive and proximate sensor modalities (with a few exceptions, such as [104]), to better understand what additional content is being provided and how best to integrate this information into existing control systems. Similarly, no significant research has been done into the timing of predictions provided by these sensor modalities and how best to capitalize on the ability of teleceptive sensors to provide advanced planning, which is not possible with proximate sensors. Finally, in addition to expanding teleceptive sensing into less explored applications such as endpoint control or mid-level activity control, other applications that would benefit from the addition of teleceptive sensors could be investigated.

## 6. Conclusions

Integration of teleceptive sensing, i.e., sensing without physical contact, into the control architectures of powered, wearable assistive devices is increasingly being considered. Several the different sensor modalities described here have become more feasible as computing capabilities have advanced over the last few decades. Several studies have described the application of these sensor modalities to both upper and lower-limb wearable assistive devices, in particular for upper-limb grasp prediction and arm endpoint control and gait event segmentation, locomotion mode forward prediction, and activity control for lower-limb devices. Although preliminary results from these studies have been promising, additional testing and verification in online, real-time, real-world scenarios is needed before translation of these technologies out of the laboratory can be safely attempted.

## Figures and Tables

**Table 1:** Teleceptive Sensing for Upper Limb Assistive and Rehabiliation Robotics.

Year	Authors	Device	Online	Modality	Teleceptive Sensor	Placed	Sensing Setup	Other Sensors	Subjects	Prediction	Processing	Accuracy
**Grasp Preshaping**												
2010	Došen and Popovic’ [66]	-	-	RGB and Ultrasound	EXOO-M053 Webcam, SRF04 Ultrasound	In Hand	320 × 240 pixels	EMG	-	Object Size	Object Segmentation	79%
2010	Došen, et al. [67]	CyberHand Prosthesis	Yes	RGB and Ultrasound	EXOO-M053 Webcam, SRF04 Ultrasound	In Hand	320 × 240 pixels	EMG	13 AB	Grasp Type, Object Size	Object Segmentation	84%
2014	Markovic, et al. [68]	SmartHand Prosthesis	Yes	Stereo RGB	Vuzix AR Headset, with Stereo RGB Cameras	Glasses	30 Hz, 640 × 480 pixels	EMG	13 AB	Grasp Type, Hand Aperture	Geometric Model	90%
2014	Gardner, et al. [70]	Bebionic v2 Hand	Yes	RGB	Logicam USB Webcam	In Hand	640 × 480 pixels	MMG	1 AB	Grasp Type	Edge Detection	84%
2015	Markovic, et al. [69]	Michelangelo Hand, Wrist	Yes	RGB-D, ToF	Creative Senz3D RGBD Camera	Glasses	30 Hz, 320 × 240 pixels	EMG, IMU	10 AB, 1 TR	Grasp Type, Wrist Orientation	Geometric Model	1 cm, 9°
2016	DeGol, et al. [72]	Slade et al Hand	Yes	RGB	PointGray Firefly MV RGB Camera	In Hand	640 × 480 pixels	-	-	Grasp Type	CNN	93%
2016	Tang, et al. [73]	iLimb Hand	Yes	RGB	Dynamic Vision Sensor	In Hand	128 × 128 pixels	-	-	Object Rotation, Class	PRST-NDIST/ CNN	96%
2017	Ghazaei, et al. [75]	iLimb Ultra, Wrist Rotator	Yes	RGB	Logitech QuickCam Chat RGB Camera	In Hand	640 × 480 pixels, to 48 × 36	-	2 TR	Grasp Type	CNN	85%
2017	Giordaniello, et al. [71]	-	-	RGB	Tobii Pro Glasses II, with RGB Camera	Glasses	25 Hz	EMG, Gaze, Cyberglove	7 AB	Grasp Type	Random Forest	75%
2017	Bu, et al. [76]	Custom Arm	Yes	RGB	RGB Images	-	256 × 256 pixels	-	1 AB	Object Class	CNN	90%
2019	Taverne, et al. [77]	-	-	RGB-D, SL	Orbbec Astra Mini S RGB-D Camera	Armband	30 Hz, 320 × 240 pixels	IMU	1 AB	Grasp Type	CNN	96%
**Endpoint Control**												
2014	Martin, et al. [78]	Custom Arm	Yes	RGB-D, Structured	Microsoft Kinect v1	Helmet	-	EMG	1 TH	Grasp Type, Object Location	Blob Detection	-
2017	Madusanka, et al. [61]	MoBio	-	RGB, Ultrasonic	-	In Hand	-	EMG	8 AB	Elbow Flex/ Extension	Visual Servoing	93%
2019	Krausz, Lamotte, et al. [62]	-	-	RGB	SMI Eye Tracking Glasses, RGB Camera	Glasses	30 Hz, 1280 × 960 pixels	EMG, Gaze, Motion Cap.	7 AB	Object Location	Corner-Based Registration	7 cm

**Table 2:** Teleceptive Sensing for Lower Limb Assistive and Rehabilitation Robotics.

Year	Authors	Device	Online	Modality	Teleceptive Sensor	Placed	Sensing Setup	Other Sensors	Subjects	Prediction	Processing	Accuracy
**Gait Segmentation**												
2018	Hu, et al. [86]	-	-	IR ToF	Camboard Pico Flexx ToF	Right Thigh	15 Hz, 171 × 224 pixels	IMU	1 AB	Heel Contact and Toe Off	Leg and Ground Segmented	6 ms
**Forward Prediction**												
2011	Zhang, et al. [87]	-	-	IR ToF Rangefinder	Leuze Electronic Optical Laser	Waist or Shank	100 Hz	IMUs	1 AB	Locomotion Activity	Distance	98%
2015	Krausz and Hargrove [91]	-	-	RGB	Google Glass RGB	Glasses	-	-	-	Stair Recognition	Edge Detection	-
2015	Krausz, et al. [90]	-	-	RGB-D, SL	Microsoft Kinect v1	Chest	5 Hz, 320 × 240 pixels	-	1 AB	Stair Recognition	Geometric Segmentation	99%
2016	Liu, et al. [88]	Custom Knee, Otto Bock Foot	Yes	IR ToF Rangefinder	Leuze Electronic Optical Laser	Waist	100 Hz	IMU, EMG, 6 DoF load Cell	6 AB, 1 TF	Locomotion Activity	Decision Tree	98%
2016	Varol, et al. [97]	-	-	RGB-D, ToF	DepthSense DS 325 RGB-D Camera	Shank	30 Hz, 320 × 240 pixels	RGB (Annotated)	1 AB	Locomotion Activity	Depth Difference Feats	99%
2018	Massalin, et al. [98]	-	-	RGB-D, ToF	DepthSense DS 325 RGB-D Camera	Shank	30 Hz, 320 × 240 pixels	RGB (Annotated)	12 AB	Locomotion Activity	Depth Difference Feats	95%
2018	Zhao, et al. [93]	-	-	RGB-D, ToF	Microsoft Kinect v2	Waist	512 × 424 pixels	IMU	-	Stair Recognition	Plane Segmentation	1 cm
2018	Kleiner, et al. [96]	BiOM Ankle	-	Radar	94 GHz FMCW Radar Sensor	Shank	160 Hz	IMU	-	Stair Height	Radial Distance	∼1 cm
2018	Yan, et al. [95]	-	-	RGB-D, SL	Xtion PRO LIVE Camera	Waist	30 Hz, 640 × 480 pixels	-	9 AB	Locomotion Activity	Hough Lines, etc.	82%
2019	Laschowski [99]	-	-	RGB	GoPro Hero4 Session	Chest	60 Hz, 1280 × 720 pixels	-	-	Locomotion Activity	CNN	95%
2019	Novo Torres, et al. [100]	-	-	RGB	ArduCam RGB Sensor Camera	Glasses	128 × 128 pixels	-	2 AB	Obstacle Recognition	CNN	90%
2019	Zhang, et al. [103]	-	-	IR ToF	Camboard Pico Flexx ToF	Thigh	15/25 Hz, 171 × 224 pixels	IMU	6 AB, 3 TF	Locomotion Activity	CNN	99%
2019	Carvalho, et al. [89]	-	-	IR ToF Rangefinder	TF Mini LiDAR Laser Sensor	Waist	100 Hz	-	10 AB	Locomotion Activity	Decision Tree	92%
2019	Khademi, et al. [101]	-	-	RGB	iPhone 8	Waist	100 Hz, 240 × 240 pixels	IMU	4 AB	Locomotion Activity	CNN	99%
2019	Krausz, et al. [104]	-	-	IR ToF	Camboard Pico Flexx ToF	Waist Belt	5 Hz, 171 × 224 pixels	IMU, Gonio, EMG	10 AB	Variability & Separability	Feature Variability	-
2019	Krausz and Hargrove [105]	OSL Knee/Ankle Prosthesis	-	IR ToF	Camboard Pico Flexx ToF	Waist Belt	15 Hz, 171 × 224 pixels	6 DoF Load Cell, IMUs	1 TF	Locomotion Activity	LDA	99%
2019	Liu, et al. [64]	VALOR Hip/Knee Exoskeleton		Stereo RGB	RealSenseD415	Waist	640 × 480 pixels	-	3 AB	Obstacle Location	Geometric Segmentation	7.5 mm
**Activity-Specific Control**												
2009	Scandaroli, et al. [63]	Knee Ankle Prosthesis	-	IR ToF Rangefinder	Sharp GP2D120 Distance IR	Under Foot	100 Hz	Gyros	-	Foot Orientation	Extended Kalman Filter	99%

## Abbreviations

The following abbreviations are used in this manuscript:

ToF	Time of Flight
SL	Structured Light
EMG	Electromyography
PID	Proportional-Integral-Derivative Controller
RGB	Red, Green, Blue
RGB-D	Red, Green, Blue-Depth
LiDAR	Light Detection and Ranging
PMD	Photonic Mixer Device
IMU	Inertial Measurement Unit
MMG	Mechanomyography
CNN	Convolutional Neural Network
SVM	Support Vector Machine
LSTM	Long-Short-Term-Memory
RANSAC	Random Sampling Consensus
AR	Augmented Reality
AB	Able-Bodied
TR	Transradial
TH	Transhumeral
TF	Transfemoral
DoF	Degree of Freedom
DVS	Dynamic Vision Sensor
FMCW	Frequency-Modulated Continuous Wave
